# Core Sets of Kinematic Variables to Consider for Evaluation of Gait Post-stroke

**DOI:** 10.3389/fnhum.2021.820104

**Published:** 2022-02-24

**Authors:** Heidi Nedergård, Lina Schelin, Dario G. Liebermann, Gudrun M. Johansson, Charlotte K. Häger

**Affiliations:** ^1^Department of Community Medicine and Rehabilitation, Physiotherapy, Umeå University, Umeå, Sweden; ^2^Department of Statistics, Umeå School of Business, Economics and Statistics, Umeå University, Umeå, Sweden; ^3^Department of Physical Therapy, Sackler Faculty of Medicine, Stanley Steyer School of Health Professions, Tel Aviv University, Tel Aviv, Israel

**Keywords:** gait analysis, walking, stroke, biomechanical evaluation, instrumented gait analysis

## Abstract

**Background:**

Instrumented gait analysis post-stroke is becoming increasingly more common in research and clinics. Although overall standardized procedures are proposed, an almost infinite number of potential variables for kinematic analysis is generated and there remains a lack of consensus regarding which are the most important for sufficient evaluation. The current aim was to identify a discriminative core set of kinematic variables for gait post-stroke.

**Methods:**

We applied a three-step process of statistical analysis on commonly used kinematic gait variables comprising the whole body, derived from 3D motion data on 31 persons post-stroke and 41 non-disabled controls. The process of identifying relevant core sets involved: (1) exclusion of variables for which there were no significant group differences; (2) systematic investigation of one, or combinations of either two, three, or four significant variables whereby each core set was evaluated using a leave-one-out cross-validation combined with logistic regression to estimate a misclassification rate (MR).

**Results:**

The best MR for one single variable was shown for the *Duration of single-support* (MR 0.10) or *Duration of 2nd double-support* (MR 0.11) *phase*, corresponding to an 89–90% probability of correctly classifying a person as post-stroke/control. Adding *Pelvis* s*agittal ROM* to either of the variables *Self-selected gait speed* or *Stride length*, alternatively adding *Ankle sagittal ROM* to the *Duration of single-stance phase*, increased the probability of correctly classifying individuals to 93–94% (MR 0.06). Combining three variables decreased the MR further to 0.04, suggesting a probability of 96% for correct classification. These core sets contained: (1) a spatial (*Stride/Step length*) or a temporal variable (*Self-selected gait speed/Stance time/Swing time* or *Duration of 2nd double-support)*, (2) *Pelvis* s*agittal ROM* or *Ankle plantarflexion during push-off*, and (3) *Arm Posture Score* or *Cadence* or a knee/shoulder joint angle variable. Adding a fourth variable did not further improve the MR.

**Conclusion:**

A core set combining a few crucial kinematic variables may sufficiently evaluate post-stroke gait and should receive more attention in rehabilitation. Our results may contribute toward a consensus on gait evaluation post-stroke, which could substantially facilitate future diagnosis and monitoring of rehabilitation progress.

## Introduction

Instrumented motion analysis is increasingly used to evaluate movement patterns during gait post-stroke (Baker et al., 2016). It serves to identify specific gait deviations based on objective information to guide clinical decision-making, individual treatment and rehabilitation. Different forms of instrumented measurement techniques, such as timing devices, pressure-sensitive walkways, sensor systems, and 3D motion capture systems, have become popular and are today often available in clinics. However, the resulting raw data is vast and may yield almost endless biomechanical descriptors. Therefore, the need for a consensus regarding which variables to focus on in evaluation and rehabilitation as the most relevant and informative descriptors of gait post-stroke has thus been pointed out repeatedly (Krasovsky and Levin, 2010; Wikström et al., 2014; Wonsetler and Bowden, 2017; Sharififar et al., 2019; Nedergård et al., 2021).

The focus of gait analysis is commonly on the lower limbs. Recent studies nevertheless emphasize the importance of incorporating also the trunk and upper limbs, particularly in post-stroke gait analyses, since this information adds to the understanding of balance control, energy expenditure and functional ability (Verheyden et al., 2006; Stephenson et al., 2010; Punt et al., 2015; Van Criekinge et al., 2017). In addition, post-stroke gait analyses that include more than the lower limbs can identify deviations in other body parts and their possible underlying causes or consequences (Kahn et al., 2019). That kind of information is important when assessing gait function, tailoring exercises that aim to improve gait post-stroke, and evaluating the effects of treatment. There is however a lack of consensus concerning which standard variables to incorporate in whole-body analyses for evaluation of gait post-stroke. This lack of agreement aggravates comparisons between research studies and may detriment the process of evaluating gait rehabilitation post-stroke (Sharififar et al., 2019; Nedergård et al., 2021). The aim of this study was hence to contribute toward such a consensus by identifying a core set of a few kinematic variables to discriminate post-stroke gait from the gait of non-disabled controls. For this purpose, we used a statistical process including leave-one-out cross-validation combined with logistic regression. Similar methods have previously been applied to other populations, e.g., in proposing a test battery for individuals with rupture of the anterior cruciate ligament (Schelin et al., 2017). In our analysis, we included the most common kinematic gait variables obtained from the literature that have been used to evaluate gait post-stroke, as well as information of the upper limb (Johansson et al., 2014) and the coordination of the upper and lower body (Nedergård et al., 2020).

## Materials and Methods

### Participants

This cross-sectional study involved 31 persons post-stroke and 41 non-disabled controls. Characteristics of the participants are presented in Table 1. The stroke group was recruited from two clinics in Umeå, Sweden. Inclusion criteria were: 35–85 years of age; > 3 months since stroke onset; unilateral hemiparesis following ischemic or hemorrhagic stroke; ability to walk indoors without aids; comprehension of written and verbal information. Exclusion criteria were impairments or diseases other than stroke which could influence gait. The post-stroke participants were assessed as having an average of moderate motor impairments (Duncan et al., 1992) with a total score of 77 ± 18 (100 maximum) on the Fugl-Meyer Assessment (Fugl-Meyer et al., 1975). The controls were recruited among colleagues, acquaintances and through a local organization for retired persons. Individuals with musculoskeletal or neurological movement impairments were excluded from the control group. Some of the participants were also included in a previous study addressing arm swing during post-stroke gait (Johansson et al., 2014). The study was approved by the Regional Ethical Review Board in Umeå, Sweden (Dnr. 2011-199-31). All participants signed a written informed consent form before participation in accordance with the Declaration of Helsinki.

**TABLE 1:** Participant characteristics (including summary information for the three explanatory variables for the first step of the analysis).

Background variables	Persons post-stroke	Controls	*p*-value
Sex (male/female)	18/13	21/20	0.74
Age, years	67.3 (10.5)	64.9 (11.5)	0.36
Body mass index*	27.6 (3.9)	24.9 (2.3)	0.00
Height, cm	171.1 (8.2)	173.9 (8.8)	0.64
Time since stroke, months	25.7 (22.1)	N/A	N/A
Brain lesion side R/L (n)	15/16	N/A	N/A
Gait speed*	0.9 (0.3)	1.3 (0.1)	0.00

*Significant differences between groups (p < 0.05). Means (standard deviations) are presented together with p-values from the respective t-test. The number of males/females is presented with a p-value from a Chi-squared test.

### Procedures

Gait analyses were performed in the U-Motion Laboratory at Umeå University, Sweden. Seventy-two reflective markers were attached to anatomical locations according to a full-body model described in detail previously (Frykberg et al., 2014). Markers were placed according to a strict protocol by three physiotherapists. Participants were instructed to walk barefoot at a self-selected speed on a 10 m walkway for a minimum of six trials. Kinematics were captured by an eight-camera three-dimensional motion capture system (240 Hz; Oqus^®^, Qualisys Gothenburg, Sweden). Data were recorded from the middle 3 m of the walkway and included 2–4 gait cycles for each trial. The data were analyzed using the Qualisys Track Manager software (QTM; Qualisys), filtered (15 Hz, fourth-order bidirectional low-pass Butterworth digital filter) and processed in Visual 3D (C-motion, Germantown, MD, United States).

### Variables and Stepwise Data Analyses

Included kinematic variables are presented in Table 2. Range of motion (ROM) was defined as the difference between the highest and lowest values of the angular joint motion curve, and the maximum (MAX) joint angle was defined as the highest value of the angular joint motion. ROM Index (ROMI) represents the ratio value of ROM, comparing the affected and non-affected side (A/NA) in persons post-stroke, and the non-dominant and dominant side (ND/D) in controls. Upper and lower body inclination angles were defined as the highest value of the inclination angle between the ankle and COM, and the head and COM, respectively (Nedergård et al., 2020). If not specified otherwise, all variables were represented by extracted discrete values based on information from the entire GC. Data from the affected side were depicted for all unilateral outcomes with the one exception of hip abduction during the stance phase. The deviation scores: Gait Profile Score, (Baker et al., 2009), Gait Deviation Index (Schwartz and Rozumalski, 2008) and the Arm Posture Score (Riad et al., 2011; Frykberg et al., 2014), represented the root mean square deviation between the lower and upper limb joint angles of each participant of the post-stroke group and the average of the controls. For further descriptions of the variables, including descriptive statistics (see Supplementary Appendix A: Tables A–C).

**TABLE 2:** Variables included in Step I of the analysis.

	Spatial and temporal gait variables	ROM joint angles	MAX joint angles	ROM Index	Lower and upper body inclination angles	Gait deviation scores	Stroke-specific gait variables
**Included**	Step length	Pelvis X	Hip Z	Hip X	A-CoMIA, stance	GPS GDI	Hip extension, swing
	Stride length	Hip X	knee X	Hip Y	A-CoMIA, swing	APS	
	Step width	Hip Y	Shoulder Y	Knee X	A-CoMIA, stance		Knee flexion, swing
	Self-selected gait speed	Hip Z		Ankle X			
	Cadence	Knee X		Shoulder X			
	Stance phase duration	Ankle X	Elbow X				Ankle plantarflexion, terminal stance
	Stance phase duration	Thorax Y					
	Duration of 1st double-support	Thorax Z					
	Duration of 2nd double-support	Shoulder X					
	Step time	Shoulder Y					
	Support	Shoulder Z					
	Swing time	Elbow X					
	Stride time						
	Temporal symmetry						
	Spatial symmetry						

**Excluded**		Thorax X	Pelvis X	Pelvis X	H-CoMIA, swing		Hip abduction, swing
		Pelvis Z	Pelvis Z	Hip Z			
		Pelvis Y	Pelvis Y	Shoulder Y			Ankle dorsiflexion, swing
			Hip X	Shoulder Z			
			Hip y				
			Ankle X				
			Shoulder X				
			Shoulder Z				Hip abduction in the non-affected side, stance
			Eibow X				Knee extension, single stance

*Spatial and temporal gait variables ROM joint angles. The variables that differed significantly between persons post-stroke and controls were included in the subsequent analysis (Step II).*

*A-CoMIA = ankle-center of mass inclination angle; APS = arm posture score; GDI = Gait Deviation Index; GPS = gait profile score; H-CoMIA = head-center of mass inclination angle MAX = maximum = sagittal plane; ROM = Range of motion; X = sagittal plane; Y= frontal plane; Z = transversal plane. If not specified, data from the affected side are depicted for all unilateral outcomes.*

The statistical approach consisting of three steps, briefly presented below, followed the main setup in Schelin et al. (2017). For all outcome variables presented in Supplementary Appendix A: Table A, we used a linear regression model to test whether there was a difference between persons post-stroke and non-disabled persons while controlling for age, sex and BMI (Step I). Descriptive statistics for the background variables age, sex and BMI are presented in Table 1. Variables with non-significant group differences were excluded from the subsequent analyses (Table 2).

In order to identify one or several potential core sets of variables, we systematically investigated all combinations consisting of 1–4 variables obtained from the complete set of all those variables that were significantly different between groups. Each core set was evaluated using leave-one-out cross-validation in combination with logistic regression to estimate the different models’ misclassification rate (MR) (Step II). For each core set, a logistic regression model based on the variables in the core set, with no interaction terms, was used to model the probability that the person (that was left out) belonged to the group of persons post-stroke. The default threshold of 0.5 was used for classification, implying that for an estimated probability above 0.5, the person was classified as post-stroke. The MR was estimated as the proportion of wrongly classified individuals. Confusion matrices that identified the rates of true positive, true negative, false positive, and false negative classifications were also considered. To add information about potential relationships between the variables, Spearman’s rank correlation was used to estimate the pair-wise correlations between the variables of interest. All analyses were performed in R (R Core Team, 2014).

## Results

The first step in the analysis process identified which variables that were significantly different between persons post-stroke and non-disabled controls (see Table 2, and with further details reported in Supplementary Appendix A: Tables A–C).

In the second step, we calculated the MR for single variables, as well as for the combination of different variables. When using only one variable, the MR ranged from 0.10 to 0.42. The lowest, hence also the best, MR (0.10) was shown for the *Duration of single-support* phase in the affected side (Table 3). This corresponds to a 90% probability of correctly classifying a person as post-stroke or control based on this specific variable. *Duration of 2nd double-support* phase classified 89% (MR 0.11), and *Stride length* 87% (MR 0.13) of the persons correctly. A core set of two variables resulted in MRs ranging from 0.06 to 0.42. The lowest rate (MR 0.06) was shown for *Step length/Stride length* in combination with *Pelvis sagittal ROM* (pelvis anterior/posterior tilt). These two combinations of variables classified 94% of persons correctly. Combining three variables decreased the MR further to 0.04 (range: 0.04–0.42). Each core set of three variables correctly classified 96% of persons. A spatial (*Stride length/Step length*) or temporal variable (*Self-selected gait speed/Stance time/Swing time/Duration of 2nd double-support*) was combined with either *Pelvis sagittal ROM or Ankle plantarflexion, terminal stance* (during push-off). In addition, these variables were combined with either *Arm Posture Score, Cadence*, or a knee or shoulder joint angle variable, to generate the best possible MR based on our populations. Adding yet another variable did not decrease the MR further, nor did it change the types of variables included in the core sets (all core sets still included at least one variable describing joint angle motions, most often *Pelvis sagittal ROM*). The combination of exclusively spatial and temporal gait variables (i.e., not including joint angle data) that most correctly classified persons post-stroke was *Stride width, Cadence, Duration of stance* phase and *Duration of 2nd double-support* phase (Table 3). This combination correctly classified 93% of persons (MR 0.07).

**TABLE 3:** The five lowest misclassification rates (MR) for one variable alone, or for core sets of 2–3 variables in combination.

	Variables			MR
**One variable**	*Duration of single-support*					0.10
	*Duration of 2nd double-support*					0.11
	*Stride length*					0.13
	*Self-selected gait speed*					0.15
	*Step length*					0.17

**Two variables**	*Step/stride length*		+	*Pelvis sagittal ROM*		0.06
	*Self-selected gait speed*		+	*Pelvis sagittal ROM*		0.07
	*Duration of single-support*		+	*Ankle sagittal ROM*		
	*Duration of single-support*		+	*Ankle plantarflexion, terminal stance*		
	*Duration of 2nd double-support*		+	*Knee flexion, swing*		

**Three variables**	*Stride/Step length*	+	*Pelvis sagittal ROM*	+	*Shoulder sagittal ROM Index/Knee sagittal ROM*	0.04
	*Self-selected gait speed*	+	*Pelvis sagittal ROM*	+	*Shoulder sagittal ROM Index/Knee flexion MAX/Arm Posture Score/Cadence*	
	*Stance/Swing time*	+	*Pelvis sagittal ROM*	+	*Knee flexion MAX*	
	*Duration of 2nd double-support*	+	*Ankle plantarflexion, terminal stance*	+	*Shoulder abduction MAX*	

**Spatial and temporal gait parameters**	*Stride width* + *Cadence* + *Duration of stance phase* + *Duration of 2nd double-support*					0.07
	*Self-selected gait speed* + *Swing time/Temporal asymmetry*					0.09

*MAX, maximum; ROM, range of motion.*

Since the MR does not include information about whether the different models are better at classifying post-stroke persons or controls, this was addressed with confusion matrices to identify the rates for true positive (0.39), true negative (0.57), false positive (0.00), and false negative (0.04) classifications. These values are valid for all core sets of size three with MR equal to 0.04. The results showed that persons post-stroke and controls were classified correctly to a similar extent in the models with low MRs.

When investigating potential relationships between the variables, correlation analyses were performed based on pooled data from both groups but also calculated separately for each group (Supplementary Appendix B: Figures A–C). The correlation matrices revealed both similarities and differences in the correlations between variables when comparing persons post-stroke and controls. In both groups, the spatial and temporal variables were generally significantly correlated, although with some exceptions (see Supplementary Appendix B: Figures B,C). Correlations for joint angle data were fewer among non-disabled controls than in persons post-stroke.

## Discussion

The stepwise analysis demonstrated that using only a single variable correctly discriminated persons post-stroke from controls with a 60–90% probability (MR 0.10-0-42) (Table 3). The most sensitive stand-alone variable was the *Duration of single-support* (MR 0.10) followed by the *Duration of 2nd double-support* (MR 0.11). Adding *Pelvis sagittal ROM* to the specific temporal or spatial variables (see Table 3) generated core sets that increased the probability of correctly classifying individuals to as high as 93–94%. Adding yet another kinematic variable to the core sets increased the probability even further to 96%.

Several of the gait variables represented in the core sets, such as the *Duration of single-* or *2nd double-stance* (longer in persons post-stroke), *Self-selected gait speed* and *Stride/Step length* (slower and shorter, respectively, in persons post-stroke), are common, well-established variables used for evaluation of gait in persons post-stroke (Balaban and Tok, 2014; Sheffler and Chae, 2015; Baker et al., 2016). As confirmed by our correlation matrices (Supplementary Appendix B: Figures A–C) and in agreement with previous research (Olney and Richards, 1996; Nadeau et al., 2013; Sharififar et al., 2019), these variables were highly interrelated and also generated similar MRs. Note, however, that *Cadence* alone received the highest MR of the spatial and temporal gait variables and only classified persons correctly with a 68% probability (MR 0.32).

Our results indicate that for improved sensitivity, spatial and temporal variables should be analyzed in combination with variables such as the *Pelvis sagittal ROM* or knee/shoulder kinematics. When used independently, several variables had a higher probability of correctly differentiating persons post-stroke from controls than *Pelvis sagittal ROM.* But when *Pelvis sagittal ROM* was used in combination with other variables, it generated improved MRs and thus seems highly relevant for post-stroke gait analysis. Indeed, two-thirds of the here presented core sets combining 2–3 variables included this specific variable. The pelvis connects the trunk to the lower limbs, and muscular control at the pelvis has been suggested to be crucial for dynamic balance and weight transmission during walking (Karthikbabu et al., 2016). Data from our post-stroke population confirm earlier observed increased ROM regarding pelvis anterior/posterior tilt among persons post-stroke (Verheyden et al., 2014; Karthikbabu et al., 2017). This is presumed to be a consequence of impaired trunk control as well as due to adaptations following lower limb impairments (Van Criekinge et al., 2017). The range of sagittal motion of the pelvis is, however, relatively small especially compared to the range of sagittal motion in the lower limbs (Chen et al., 2003). This makes pelvic tilt more difficult to observe or measure in clinical settings without any technological devices. Furthermore, the constitution and attire of an individual may complicate assessing the pelvic tilt during gait. Nevertheless, the pelvic function should be considered when evaluating and treating gait post-stroke. Selective pelvic exercises, for instance, seems to have a beneficial effect on trunk function, standing balance, and mobility after stroke (Haruyama et al., 2016).

Altered knee movement patterns, such as greater or lesser knee flexion during the early stance phase followed by knee hyperextension in the late stance phase, have been reported post-stroke (Woolley, 2001; Baker et al., 2016). For the swing phase, previous studies have shown that the most common knee joint angle deviation is a generally decreased knee flexion and/or decreased knee extension prior to initial contact (Olney and Richards, 1996; Balaban and Tok, 2014; Baker et al., 2016). In 3D gait analysis, the knee sagittal kinematics are considered as reliable variables that also are suggested to predict gait function post-stroke (Guzik et al., 2020). In agreement with that, our results suggest that sagittal knee angle motions (represented by *Knee flexion MAX* and *Knee sagittal ROM*) are of particular importance when classifying gait post-stroke. These two variables were represented in several emerging core sets. For the *Knee sagittal ROM*, estimates of the minimal detectable change and the minimal clinically important difference have been established for persons post-stroke (Geiger et al., 2019; Guzik et al., 2020) and these estimated values are useful when interpreting changes of knee flexion/extension observed in persons who have undergone gait interventions post-stroke.

In contrast to the focus on the lower limbs, kinematics of the upper limbs during gait post-stroke has been far from as extensively investigated (Carmo et al., 2012). A few recent studies have, however, emphasized the importance of incorporating the trunk (i.e., the thorax) and arm movements in post-stroke gait analyses (Stephenson et al., 2010; Carmo et al., 2012). Stroke affects upper limb function in 50–70% of persons in the subacute phase post-stroke (Krakauer, 2005) and may have a clear influence on gait (Johansson et al., 2014). In the long term, around 40% experience disabilities of the upper limbs (Alt Murphy and Häger, 2015), whereas a lower percentage present with disabilities for functions of the lower limbs. It thus becomes clear that the inclusion of upper limb kinematic variables is of importance for ensuring comprehensive gait analyses. In the current study, upper limb motions (*Shoulder abduction MAX, Arm Posture Score* and *Shoulder sagittal ROM Index)* were represented in several suggested core sets of variables, providing further evidence that the upper limbs influence post-stroke gait movement pattern. An increased shoulder abduction angle among persons post-stroke (*Shoulder abduction MAX)* seems characteristic for post-stroke gait and has been assumed to be part of a compensatory strategy to help improve body weight distribution and balance (Carmo et al., 2012). In addition, an earlier reported variation in shoulder movement deviations in the sagittal plane (Kahn et al., 2019) was also observed among our persons post-stroke. While some persons display excessive movements (large ROM), others employ a more fixed joint posture (small ROM) during walking. Another interesting aspect was the generally greater number of associations between joint position variables among persons post-stroke when compared with controls (see the correlation matrices, Supplementary Appendix A: Tables B,C). Even though the absence of impairments would theoretically allow for greater variation, walking on an even surface without any obstacles and in a controlled fashion seems to generate a stable movement pattern. The fewer correlations between several variables among non-disabled controls when compared to persons post-stroke may hence be explained, at least partly, by lesser variability.

The potential for assessing the core set variables in a clinical context differs. The spatial and temporal variables are most accessible in clinics today since this data can be gathered relatively efficiently with the use of, e.g., pressure-sensitive walkways or timing devices. Self-selected gait speed is one of the most commonly used outcome measures. By combining *Self-selected gait speed* (MR 0.15) in the present study with another temporal variable, such as *Duration of swing* or *Temporal asymmetry*, the MR improved to 0.09. The combination of spatial and temporal variables that most correctly seems to classify persons post-stroke and controls included *Stride width, Cadence, Duration of stance* and *Duration of 2nd double-support*. Together these four variables classified persons correctly with a 93% probability.

Gathering joint position data, compared with spatial and temporal variables, generally requires more time, equipment and data processing. For example, calculation of the *Arm Posture Score* is currently based on comprehensive 3D upper limb joint position data, whereas estimating the *Sagittal ROM Shoulder Index* requires bilateral shoulder flexion/extension data. Low-cost webcam technology has, however, shown potential for real-time tracking of joint kinematics, particularly in the sagittal plane and if performed following specific recommendations related to data collection setup and camera and analysis software (Michelini et al., 2020). Such technology opens up new, more accessible possibilities for kinematic assessments during gait which may be more easily utilized in a clinical setting compared to laboratory-based data collection with 3D motion capture systems. Data collection of *Pelvis sagittal ROM* or *Knee flexion MAX/Knee sagittal ROM*, for instance, can also be made with 2D data-collecting systems. The use of portable inertial sensors to capture kinematic information over an extended space and time and in real-life conditions has also been described by Petraglia et al. (2019) and van Schaik and Dominici (2020).

### Methodological Considerations

The purpose of Step II of the current analytical approach was to identify potential core sets of variables for discussion and future research toward a consensus on crucial variables for the kinematic evaluation of gait post-stroke. The focus of the current paper was not on checking model assumptions, interpreting parameter estimates, or comparing models in other ways than their classification ability. If identified core sets are to be used further in similar analyses, these assumptions should be checked. One specific assumption relates to the lack of multicollinearity, i.e., that the variables included in the model are not highly correlated. Note that gait speed and cadence were identified in the same core set and that these variables seem significantly correlated both in our analyses (see correlation matrices, Supplementary Appendix B: Figures A–C) and in previous research (Sharififar et al., 2019). Combining these two variables may therefore not be advisable if they represent the same underlying central nervous networks in the motor control process related to gait. While we have not *a priori* eliminated collinear variables based on conceptual conjectures, we are aware that this could result in redundancy in the discrimination process (one could potentially use various combinations that provide similar results).

Analyzing individuals walking at a self-selected speed as opposed to at their fastest possible gait speed or at a predetermined pace, will likely influence the results. Earlier research suggests, for instance, that gait speed affects the pelvic motions of non-disabled individuals (Lewis et al., 2017), while knee kinematics in the sagittal plane, on the other hand, seems not to be speed dependent when comparing persons post-stroke with non-disabled persons walking with matched gait speed (Chen et al., 2005). Due to close relationships between gait speed and other temporal and spatial variables, while persons post-stroke walked slower than non-disabled controls in our study, the core sets presented may be influenced either by the pathology *per se* or by gait speed, or both.

The generalizability of this study is limited to a population in a long-term phase post-stroke and with mainly relatively mild impairments. Previous studies have shown that e.g., pelvic tilt may vary depending on stroke onset (Van Criekinge et al., 2017) and severity level (Chen et al., 2003). Although our stroke sample does not fully represent the whole range of stroke severity, it is an important subpopulation as the individuals included may have treatable gait deficits that are not easily or clearly identified without instrumented motion analysis. Furthermore, the analysis has not considered the possible impact of the localization of injury which may affect functional outcomes and movement strategies post-stroke.

Only significant variables were included in the latter step of the analysis. Some variables could nonetheless still be important but were perhaps not significant due to lack of power. If altering the default threshold of 0.5 that was used for classification, the MR, as well as the rate of true positive/negative, might change. However, since persons post-stroke and controls both were correctly classified, and the MR was low for many models, we see only a limited gain of finding and using an optimal threshold.

Finally, almost an endless number of variables are available from instrumented gait analysis. We chose to initially include those that were considered relevant from a clinical perspective as well as the ones most commonly used, based on the literature in the field. The identified core sets contain spatiotemporal gait variables and joint motion variables in the sagittal plane, several of which are considered highly reliable in persons post-stroke and where previous analyses include estimates of minimal detectable change (Geiger et al., 2019).

## Conclusion

Our results contribute toward a consensus on which kinematic variables should be included in the evaluation of gait post-stroke. This may substantially facilitate future diagnostics and treatment planning regarding specific gait deficits post-stroke, as well as the monitoring and evaluation of rehabilitation progress in the clinics. It would also be of great value for comparisons across studies and in meta-analysis contexts if the same variables are to be used in research.

## Data Availability Statement

The raw data supporting the conclusions of this article will be made available by the authors, without undue reservation.

## Ethics Statement

The studies involving human participants were reviewed and approved by the Regional Ethical Review Board in Umeå, Sweden (Dnr. 2011-199-31). The patients/participants provided their written informed consent to participate in this study.

## Author Contributions

HN, DL, CH, and GJ formulated the research question. CH and LS obtained the funding. HN and GJ performed the data collection and data preparations. LS performed the quantitative data analysis. HN drafted the preliminary manuscript. All authors helped in further writing and critical reviewing of the manuscript, read, and approved the final manuscript.

## Conflict of Interest

The authors declare that the research was conducted in the absence of any commercial or financial relationships that could be construed as a potential conflict of interest.

## Publisher’s Note

All claims expressed in this article are solely those of the authors and do not necessarily represent those of their affiliated organizations, or those of the publisher, the editors and the reviewers. Any product that may be evaluated in this article, or claim that may be made by its manufacturer, is not guaranteed or endorsed by the publisher.
