# Supplementary material for: Core Sets of Kinematic Variables to Consider for Evaluation of Gait Post-stroke
Source: Front Hum Neurosci. 2022 Feb 24;15:820104. doi: 10.3389/fnhum.2021.820104 (PMC8908020; doi:10.3389/fnhum.2021.820104)
Supplement: Supplementary file 1 [file Data_Sheet_1.PDF]

Appendix B.

Figure A A-C. Correlation matrices illustrating the relationships between variables when combining data from the groups (A), and for the group of persons post-stroke (B) and controls (C), respectively. Spearman's rank correlation was used to estimate the pair-wise correlations between the variables, and only significant correlations (on significance level 0.05) are displayed in colour in Figure A-C.

A. Pooled persons post-stroke and controls

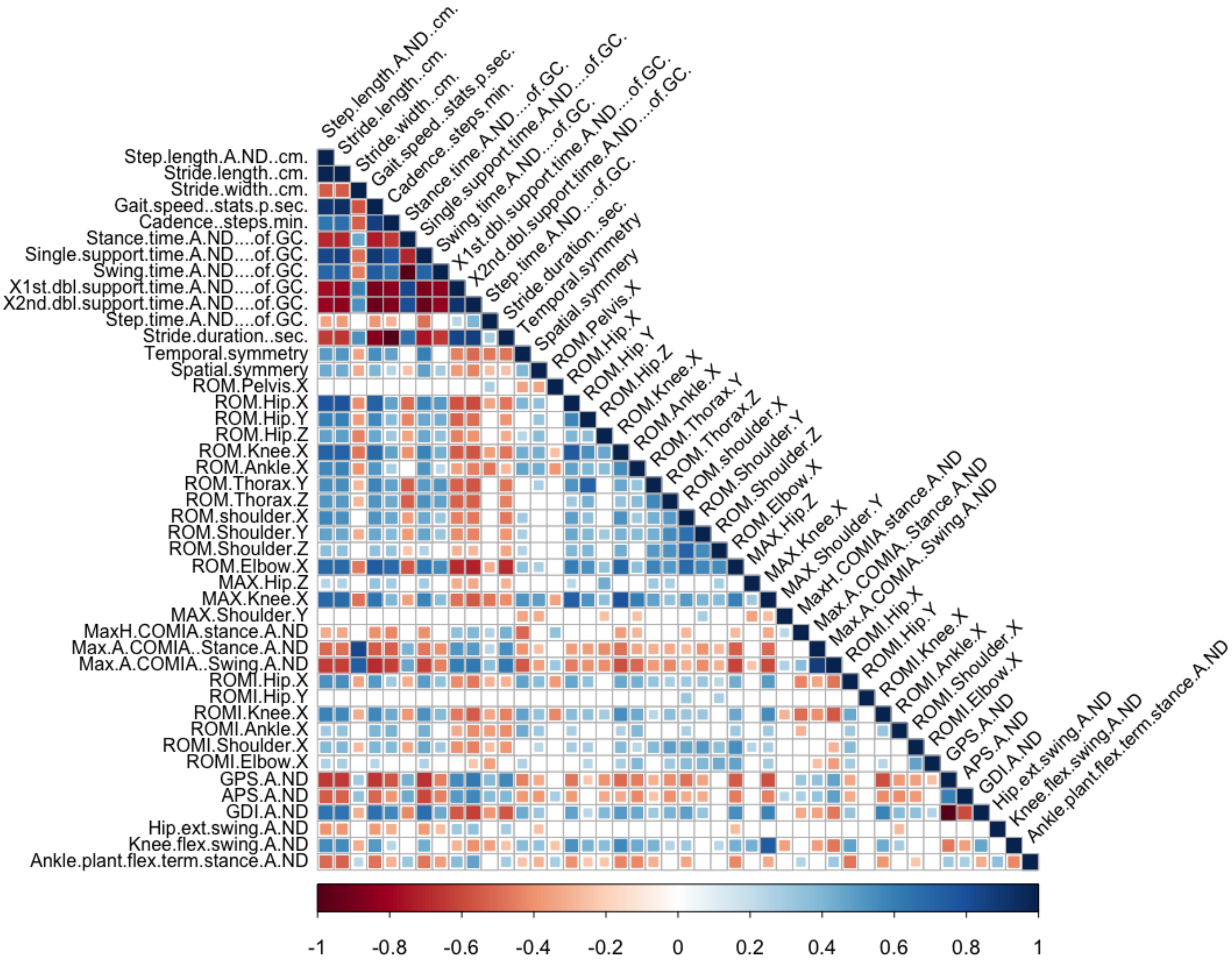

B. Persons post-stroke

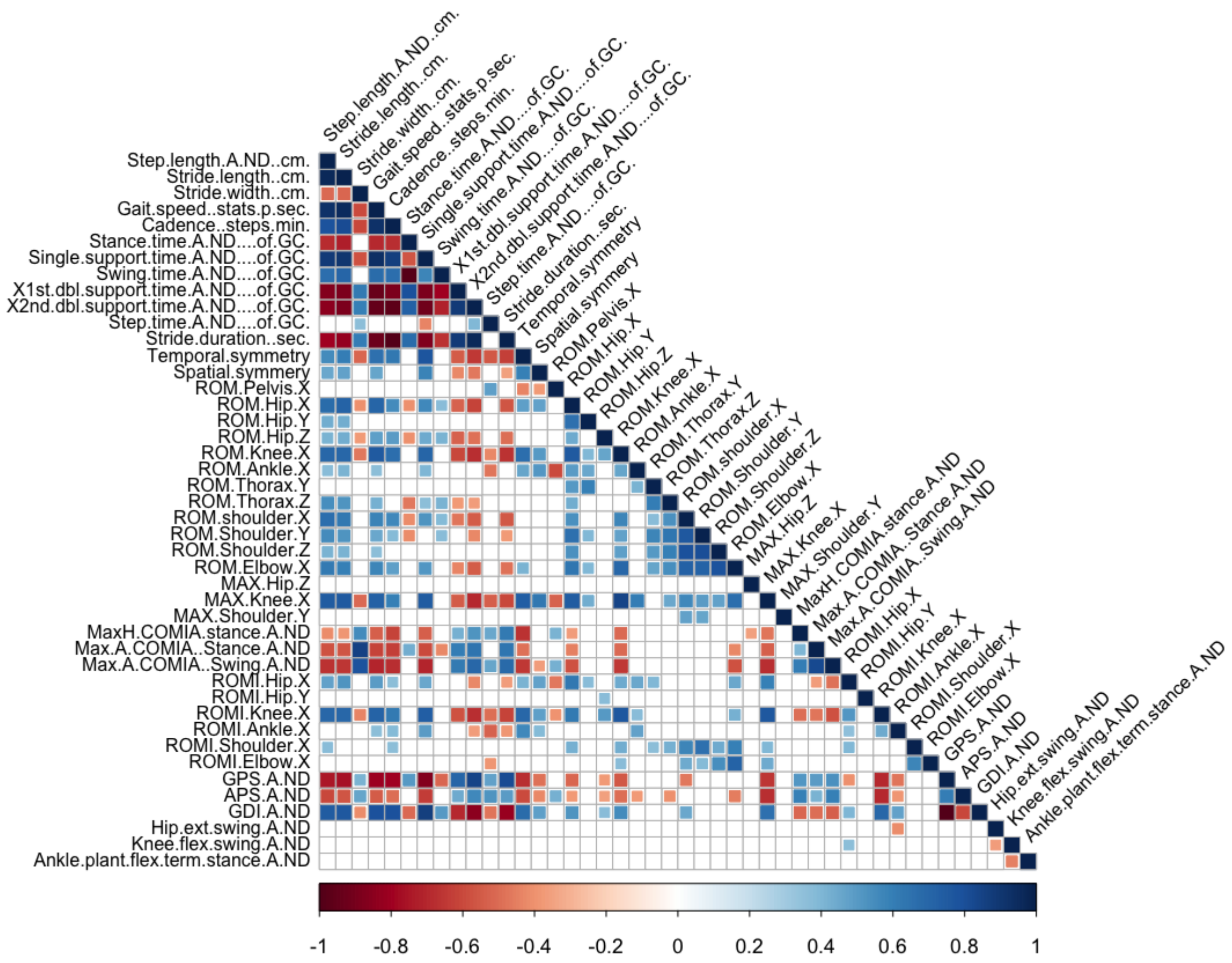

C. Controls

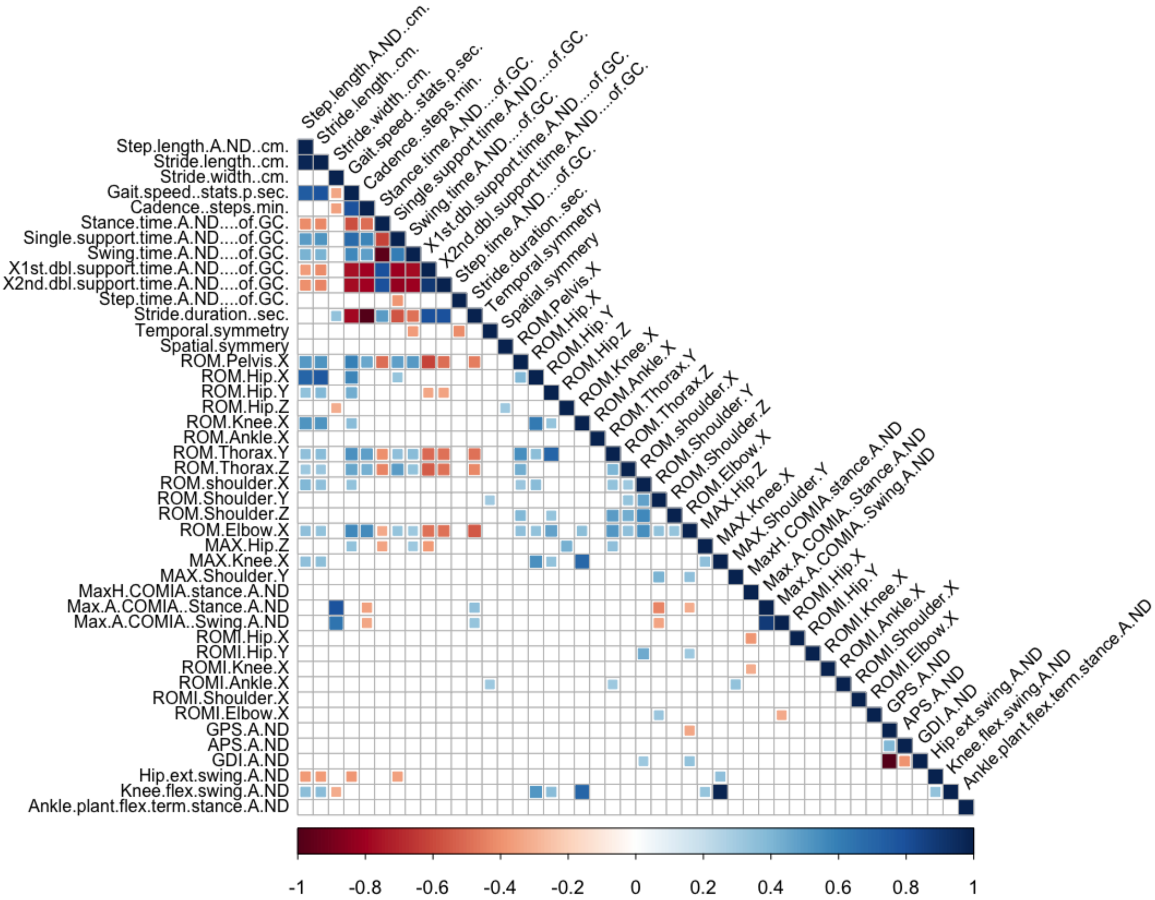

Step.length.A.ND..cm.
Stride.length..cm.
Stride.width..cm.
Gait.speed..stats.p.sec.
Cadence..steps.min.
Stance.time.A.ND....of.GC.
Single.support.time.A.ND....of.GC.
Swing.time.A.ND....of.GC.
X1st.dbl.support.time.A.ND....of.GC.
X2nd.dbl.support.time.A.ND....of.GC.
Step.time.A.ND....of.GC.
Stride.duration..sec.
Temporal.symmetry
Spatial.symmery
ROM.Pelvis.X
ROM.Hip.X
ROM.Hip.Y
ROM.Hip.Z
ROM.Knee.X
ROM.Ankle.X
ROM.Thorax.Y
ROM.Thorax.Z
ROM.shoulder.X
ROM.Shoulder.Y
ROM.Shoulder.Z
ROM.Elbow.X
MAX.Hip.Z
MAX.Knee.X
MAX.Shoulder.Y
MaxH.COMIA.stance.A.ND
Max.A.COMIA..Stance.A.ND
Max.A.COMIA..Swing.A.ND
ROMI.Hip.X
ROMI.Hip.Y
ROMI.Knee.X
ROMI.Ankle.X
ROMI.Shoulder.X
ROMI.Elbow.X
GPS.A.ND
APS.A.ND
GDI.A.ND
Hip.ext.swing.A.ND
Knee.flex.swing.A.ND
Ankle.plant.flex.term.stance.A.ND
-1
-0.8
-0.6
-0.4
-0.2
0
0.2
0.4
0.6
0.8
1
