# Supplementary material for: Core Sets of Kinematic Variables to Consider for Evaluation of Gait Post-stroke
Source: Front Hum Neurosci. 2022 Feb 24;15:820104. doi: 10.3389/fnhum.2021.820104 (PMC8908020; doi:10.3389/fnhum.2021.820104)
Supplement: Supplementary file 2 [file Table_1.DOCX]

**APPENDIX A**

**Table A.** Presentation of the variables included in Step I of the analysis

| **Variables** |  | | **Short description** |
| --- | --- | --- | --- |
| **Spatial and temporal parameters** | | |  |
|  | Step length | | toe off => initial contact, (cm) |
|  | Stride length | | initial contact => initial contact on the same leg (cm) |
|  | Stride width | | the distance between the heels of the two feet during double stance (cm) |
|  | Gait speed | | velocity normalised to height; statures per second |
|  | Cadence | | steps per minute |
|  | Duration of single-support | | opposite toe-off => opposite initial contact; % of GC; A or ND |
|  | Duration of stance phase | | initial contact => toe off; % of GC; A or ND |
|  | Duration of swing phase | | toe-off => initial contact; % of GC; A or ND |
|  | Duration of 1st double-support | | initial contact => opposite toe-off; % of GC; A or ND |
|  | Duration of 2nd double-support | | opposite initial contact => toe-off; % of GC; A or ND |
|  | Step duration | | % of GC; A or ND side |
|  | Stride time | | seconds |
|  | Temporal symmetry | | The ratio value; A/NA or ND/D |
|  | Spatial symmetry | | The ratio value; A/NA or ND/D |
| **Range of angular motion (ROM);** The difference between the highest and lowest values of the angular joint motion curve during the GG; A/ND | | | |
| Sagittal plane | Elbow | | flexion/extension |
|  | Shoulder | | flexion/extension |
|  | Thorax | | flexion/extension |
|  | Pelvis | | anterior/posterior tilt |
|  | Hip | | flexion/extension |
|  | Knee | | flexion/extension |
|  | Ankle | | flexion/extension |
| Frontal plane | Shoulder | | abduction/adduction |
|  | Thorax | | abduction/adduction |
|  | Pelvis | | Lateral tilt |
|  | Hip | | abduction/adduction |
| Transversal plane | Shoulder | | external/internal rotation |
|  | Thorax | | external/internal rotation |
|  | Pelvis | | external/internal rotation |
|  | Hip | | external/internal rotation |
| *Exclusion from further analyses because of non-significant differences between groups (p > 0.05). A = affected body side in persons post-stroke; D = dominant body side in controls; GC = gait cycle; NA = non-affected body side in controls; ND = non-dominant body side in controls. | | | |
| **Variables** | | | **Short description** |
| **Range of angular motion Index (ROMI);** The difference between the highest and lowest values of the angular joint motion curve during the GC and comparison between body sides. Ratio value: A/NA or ND/D. | | | |
| Sagittal plane | Elbow | | flexion/extension |
|  | Shoulder | | flexion/extension |
|  | Thorax | | flexion/extension |
|  | Pelvis | | anterior/posterior tilt |
|  | Hip | | flexion/extension |
|  | Knee | | flexion/extension |
|  | Ankle | | flexion/extension |
| Frontal plane | Shoulder | | abduction/adduction |
|  | Thorax | | abduction/adduction |
|  | Pelvis | |  |
|  | Hip | | abduction/adduction |
| Transversal plane | Shoulder | | external/internal rotation |
|  | Thorax | | external/internal rotation |
|  | Pelvis | |  |
|  | Hip | | external/internal rotation |
| **Maximum joint angle (MAX);** The highest value of the angular joint motion during the GC; A/ND. | | | |
| Sagittal plane | Elbow | | flexion/extension |
|  | Shoulder | | flexion/extension |
|  | Pelvis | | anterior/posterior tilt |
|  | Hip | | flexion/extension |
|  | Knee | | flexion/extension |
|  | Ankle | | flexion/extension |
| Frontal plane | Shoulder | | abduction/adduction |
|  | Pelvis | |  |
|  | Hip | | abduction/adduction |
| Transversal plane | Shoulder | | external/internal rotation |
|  | Pelvis | |  |
|  | Hip | | external/internal rotation |
| **Inclination angles;** The highest value of upper or lower body inclination angle during the stance or swing phase of the GC; A/ND. | | | |
| A-CoMIA, stance | | Lower body inclination angle defined as the angle between the estimated CoM and the mid-ankle | |
| A-CoMIA, swing | | Lower body inclination angle defined as the angle between the estimated CoM and the mid-ankle | |
| H-CoMIA, stance | | Upper body inclination angle defined as the angle between the estimated CoM and the frontal head marker | |
| H-CoMIA, swing | | Upper body inclination angle defined as the angle between the estimated CoM and the frontal head marker | |
| *Exclusion from further analyses because of non-significant differences between groups (p > 0.05). A = affected body side in persons post-stroke; A-CoMIA = ankle-CoM inclination angle; CoM = center of mass; D = dominant body side in controls; GC = gait cycle; H-CoMIA = head-CoM inclination angle; NA = non-affected body side in controls; ND = non-dominant body side in controls. | | | |
| **Variables** | | **Short description** | |
| **Deviation scores;** The root mean square deviation between the joint angles of each subject and the average of the controls during a GC | | | |
| Gait Profile Score (GPS) | | 9 GVS: pelvis tilt, obliquity, and rotation; hip flexion, abduction and rotation; knee flexion; ankle dorsiflexion; and foot progression. | |
| Arm Posture Score (APS) | | 6 GVS values based on upper limb kinematics (shoulder flexion, abduction and rotation; elbow flexion; pronation; and wrist flexion | |
| Gait Deviation Index (GDI) | | Identifies a scaled distance between 15 lower limb and trunk gait feature scores for a subject and the average of the same 15 gait feature scores for a control group. | |
| **Stroke-specific parameters;** Joint position angles that are suggested to specifically characterise post-stroke gait movement pattern | | | |
| Hip extension, swing | | Highest value of hip extension in A/ND side during the initial swing | |
| Hip abduction, swing | | Highest value of hip abduction in A/ND side during the swing phase (circumduction) | |
| Hip abduction, stance | | Highest value of hip abduction in NA/D side during the stance phase (hip hiking) | |
| Knee flexion, swing | | Highest value of knee flexion in A/ND side during the swing phase | |
| Knee extension, single-stance | | Highest value of knee extension in A/ND side during the single stance phase (knee overextension) | |
| Ankle plantarflexion, terminal stance | | Highest value of ankle plantarflexion in A/ND side during push-off | |
| Ankle dorsiflexion, swing | | Highest value of ankle dorsiflexion during the swing phase | |
| *Exclusion from further analyses because of non-significant differences between groups. A = affected body side in persons post-stroke; D = dominant body side in controls; GC = gait cycle; GVS = gait variable score; NA = non-affected body side in controls; ND = non-dominant body side in controls | | | |

| **Table B**. Detailed results for variables with significant group differences. Mean (standard deviation) for all variables are presented together with p-values for the group indicator from a linear regression model also including the explanatory variables age, sex and BMI. P-values below 0.005 are reported as 0.00. | | | | | |
| --- | --- | --- | --- | --- | --- |
|  | | | | | |
| **Biomechanical variables** | | **Post-stroke** Mean (SD) | | **Controls** Mean (SD) | **p-value** |
| **Spatial and temporal** | | | | | |
| Step length, A/ND (cm) | | 29.5 (7.9) | | 39.2 (2.9) | 0.00 |
| Stride length (cm) | | 58.8 (15.3) | | 78.5 (5.3) | 0.00 |
| Stride width (cm) | | 8.8 (2.4) | | 7.0 (1.3) | 0.00 |
| Gait speed (statures per second) | | 0.5 (0.2) | | 0.8 (0.1) | 0.00 |
| Cadence (steps/min) | | 50.3 (8.5) | | 58.7 (4.3) | 0.00 |
| Duration of stance phase, A/ND (% of GC) | | 63.8 (5.0) | | 60.0 (1.2) | 0.00 |
| Duration of single-support, A/ND    (% of GC) | | 33.1 (6.7) | | 39.6 (1.3) | 0.00 |
| Duration of swing phase, A/ND (% of GC) | | 36.3 (4.7) | | 40.1 (1.2) | 0.00 |
| Duration of 1^st^ dbl support, A/ND (% of GC) | | 0.2 (0.2) | | 0.1 (0.0) | 0.00 |
| Duration of 2^nd^ dbl support, A/ND (% of GC) | | 0.2 (0.2) | | 0.1 (0.0) | 0.00 |
| Duration of step, A/ND (% of GC) | | 52.0 (4.3) | | 50.2 (0.6) | 0.03 |
| Stride/GC duration (sec) | | 1.2 (0.3) | | 1.0 (0.1) | 0.00 |
| Temporal symmetry | | 0.9 (0.1) | | 1.0 (0.0) | 0.00 |
| Spatial symmetry | | 0.9 (0.1) | | 1.0 (0.0) | 0.00 |
| **ROM joint angles** | | | | | |
| ROM.Pelvis X | | 5.6 (2.7) | | 3.7 (0.8) | 0.00 |
| ROM.Hip X | | 39.6 (9.8) | | 47.7 (4.6) | 0.00 |
| ROM.Hip Y | | 8.3 (3.2) | | 10.5 (2.8) | 0.00 |
| ROM.Hip Z | | 15.5 (4.6) | | 18.7 (4.0.) | 0.02 |
| ROM.Knee X | | 48.7 (13.3) | | 60.8 (5.0) | 0.00 |
| ROM.Ankle X | | 20.0 (5.0) | | 25.4 (4.4) | 0.00 |
| ROM.Thorax Y | | 4.6 (2.0) | | 6.7 (2.4) | 0.00 |
| ROM.Thorax Z | | 8.9 (3.2) | | 11.5 (3.3) | 0.00 |
| ROM.Shoulder X | | 19.6 (12.8) | | 27.9 (9.7) | 0.00 |
| ROM.Shoulder Y | | 8.6 (4.5) | | 10.8 (3.4) | 0.04 |
| ROM.Shoulder Z | | 15.9 (8.7) | | 18.9 (6.5) | 0.04 |
| ROM.Elbow X | | 17.1 (12.4) | | 33.2 (12.0) | 0.00 |
| **MAX joint angles** | | | | | |
| MAX.Hip Z | | 2.7 (7.9) | | 6.9 (5.9) | 0.04 |
| MAX.Knee X | | 51.6 (13.4) | | 64.0 (4.8) | 0.00 |
| MAX.Shoulder Y | | 19.9 (6.1) | | 15.8 (2.9) | 0.03 |
| **ROM index** | | | | | |
| ROMI.Hip X | | 0.8 (0.2) | | 1.0 (0.0) | 0.00 |
| ROMI.Hip Y | | 0.8 (0.2) | | 0.9 (0.1) | 0.03 |
| ROMI.Knee X | | 0.8 (0.2) | | 1.0 (0.0) | 0.00 |
| ROMI.Ankle X | | 0.8 (0.2) | | 0.9 (0.1) | 0.00 |
| ROMI.Shoulder X | | 0.6 (0.2) | | 0.8 (0.2) | 0.00 |
| ROMI.Elbow X | | 0.6 (0.3) | | 0.8 (0.2) | 0.02 |
| **Inclination angles** | | | | | |
| Max H-CoMIA, stance.A/ND | | 3.3 (1.9) | | 1.8 (1.3) | 0.00 |
| Max A-CoMIA, stance.A/ND | | 6.6 (1.9) | | 5.3 (0.9) | 0.00 |
| Max A-COMIA, swing.A/ND | | 8.0 (2.3) | | 6.1 (0.8) | 0.00 |
| **Deviation score** | | | | | |
| GPS.A/ND | | 7.2 (2.9) | | 4.5 (1.1) | 0.00 |
| APS.A/ND | | 13.4 (5.9) | | 7.9 (2.8) | 0.00 |
| GDI.A/ND | | 81.6 (18.0) | | 101.0 (11.4) | 0.00 |
| **Stroke-specific variables** | | | | | |
| Hip extension. swing, A/ND | | 2.5 (9.2) | | -2.3 (6.4) | 0.01 |
| Knee flexion, swing, A/ND | | 51.5 (13.4) | | 64.1 (4.7) | 0.00 |
| Ankle plantarflexion terminal stance, A/ND | | -2.0 (5.5) | | -9.1 (5.2) | 0.00 |
| **Table C**. Detailed results for variables without significant group differences. Mean (standard deviation) for all variables are presented together with p-values for the group indicator from a linear regression model also including the explanatory variables age, sex and BMI. | | | | | |
| **Biomechanical variables** | **Post-stroke** Mean (SD) | | **Controls** Mean (SD) | | **p-value** |
| **ROM joint angles** | | | | | |
| ROM.Thorax X | 3.7 (1.9) | | 3.8 (1.1) | | 0.21 |
| **MAX joint angles** |  | |  | |  |
| MAX.Pelvis X | 7.9 (6.5) | | 7.3 (4.8) | | 0.76 |
| MAX.Pelvis Y | 2.5 (2.5) | | 1.8 (1.8) | | 0.22 |
| MAX.Pelvis Z | 2.4 (4.6) | | 4.3 (2.7) | | 0.19 |
| MAX.Hip X | 30.7 (9.1) | | 34.1 (6.3) | | 0.08 |
| MAX.Hip Y | 7.0 (4.0) | | 7.4 (4.0) | | 0.05 |
| MAX.Ankle X | 14.5 (3.7) | | 13.8 (2.8) | | 0.42 |
| MAX.Shoulder X | 4.2 (8.7) | | 6.5 (8.2) | | 0.14 |
| MAX.Shoulder Z | 26.9 (14.5) | | 29.0 (11.0) | | 0.76 |
| MAX.Elbow X | 56.0 (13.5) | | 54.1 (9.3) | | 0.27 |
| **ROM index** | | | | | |
| ROMI.Pelvis X | 0.9 (0.1) | | 0.9 (0.1) | | 0.20 |
| ROMI.Hip Z | 0.8 (0.1) | | 0.9 (0.1) | | 0.10 |
| ROMI.Shoulder Y | 0.7 (0.2) | | 0.7 (0.2) | | 0.24 |
| ROMI.Shoulder Z | 0.6 (0.2) | | 0.7 (0.2) | | 0.07 |
| **Body inclination angles** | | | | | |
| Max H-CoMIA, swing, A/ND | 1.9 (0.9) | | 1.7 (1.0) | | 0.30 |
| **Stroke specific variables** | | | | | |
| Hip abduction, swing, A/ND | 3.5 (4.0) | | 2.0 (3.4) | | 0.50 |
| Ankle dorsiflexion, swing, A/ND | 9.0 (4.8) | | 9.3 (2.6) | | 1.00 |
| Hip abduction, stance, NA/D | 3.7 (4.7) | | 3.7 (3.3) | | 0.69 |
| Knee extension, single-stance, A/ND | 4.3 (8.9) | | 4.2 (4.1) | | 0.52 |
